# Supplementary material for: Parents’ perspectives on a national child oral health promotion program: Sociodemographic influences and behavioral insights – A cross-sectional analysis
Source: PLoS One. 2026 Feb 27;21(2):e0334203. doi: 10.1371/journal.pone.0334203 (PMC12948110; doi:10.1371/journal.pone.0334203)
Supplement: S1 File — (DOCX) [file pone.0334203.s001.docx]

**Here’s the English translation of your provided text, keeping it formal and aligned with research questionnaire style:**

**(Questionnaire for Assessing Parents’ Perspectives on the Student Oral Health Transformation Plan)**

**“Introduction”
The Student Oral Health Promotion Program was launched in early 2015 (1394 in the Iranian calendar) through collaboration between the Oral Health Department of the Ministry of Health and the Ministry of Education, with the goal of preventing and reducing dental caries among students. Subsequently, in the 2016–2017 academic year, the program was expanded nationwide to all primary schools.**

**This program consists of four components: (1) oral health education, (2) completion of the electronic oral health record, (3) provision of preventive services (fluoride varnish application), and (4) assessment of treatment needs.**

**By evaluating these four stages, a general assessment of parents’ perspectives on this national program can be obtained.**

**“Notes”**

1. **The questionnaire is completed through telephone interviews with parents.**
2. **At the beginning of each interview, parents are informed about the importance and necessity of the program.**
3. **Parents are not obligated to complete the questionnaire.**
4. **At the beginning of the questionnaire, basic demographic information about the student and family is collected.**
5. **Some questions are designed with the options “Yes,” “No,” or “No opinion,” while others are open-ended.**
6. **The questionnaire consists of six sections: introduction to the program, notes, initial information, student and parent information, and main questions.**

**“Initial Information”**

1. **Form number: ..................**
2. **Date of completion: ....... / ....... / 139....**
3. **City: ............................
   Residential area: ..................................
   School name: ............................
   Current educational grade of the student: ..................................**

**“Information about the Student and Parents”**

1. **Relationship to student: (Mother / Father / Other)**
2. **Occupation of household head: (Government employee / Government worker / Private-sector employee / Private-sector worker / Self-employed / Homemaker / Retired / Unemployed)**
3. **Father’s education level: (Illiterate / Literate [can read and write] / Primary school / Middle school / High school diploma / Associate degree / Bachelor’s / Master’s / Doctorate or higher / Don’t know)**
4. **Number of household members: ...............**
5. **Mother’s education level: (Illiterate / Literate [can read and write] / Primary school / Middle school / High school diploma / Associate degree / Bachelor’s / Master’s / Doctorate or higher / Don’t know)**

**Main Questionnaire Questions**
Dear Parents, please answer the following questions:

Q1. **Fluoride Therapy Consent**
Did you consent to fluoride therapy for your child at school?

1. Yes □
2. No □ (Reason)
3. Other □

Q2. Were you aware that an electronic oral health profile was created for your child at school?

1. Yes □
2. No □
3. No opinion □

Q3. Were you satisfied with the electronic oral health profile created for your child in the program?

1. Yes □
2. No □
3. No opinion □

Q4. Were you aware that fluoride varnish was applied for your child at school?

1. Yes □
2. No □
3. No opinion □

Q5. Were you satisfied with the fluoride varnish therapy your child received at school?

1. Yes □
2. No □
3. No opinion □

Q6. Are you aware that your child received oral health education in the program??

1. Yes □
2. No □
3. No opinion □

Q7. Were you satisfied with the oral health education your child received in the program?

1. Yes □
2. No □
3. No opinion □

Q8. Were you aware that the program identified your child's dental treatment needs?

1. Yes □
2. No □
3. No opinion □

Q9. Were you satisfied with the dental treatment needs identified for your child in the program?

1. Yes □
2. No □
3. No opinion □

Q10. How often does your child brush their teeth per day now that the program has ended for them?

1. Twice or more □
2. Once □
3. Less than once (irregular) □
4. Never □

Q11. Does your child use fluoride toothpaste when tooth-brushing now that the program has ended for them??

1. Yes □
2. No □
3. No opinion □

Q12. How often does your child consume sugary snacks now that the program has ended for them??

1. Once or more □
2. Less than once □
3. Never □

Q13. Do you think the program has positively impacted your child's tooth-brushing habit?

1. Yes □
2. No □
3. No opinion □

Q14. What type of foods does your child typically eat at school now that the program has ended for them? Please list them below.
