# Supplementary material for: Parents’ perspectives on a national child oral health promotion program: Sociodemographic influences and behavioral insights – A cross-sectional analysis
Source: PLoS One. 2026 Feb 27;21(2):e0334203. doi: 10.1371/journal.pone.0334203 (PMC12948110; doi:10.1371/journal.pone.0334203)
Supplement: Attachment — Submitted filename: Point-by-point Response.docx [file pone.0334203.s003.docx]

**Point-by-point Response to Editor and Reviewers**

Dear Editor,

Thank you for the opportunity to revise our manuscript. We appreciate the constructive feedback which has helped us tremendously strengthen the paper. Below, we provide a point-by-point response to all comments, detailing how we addressed each one and explaining the changes made. All revisions are based on a careful review of the comments and the original manuscript. We have incorporated changes to improve clarity, adherence to journal guidelines, methodological rigor, and overall flow. The revised manuscript reflects these updates.

**Editor Comments**

*1. Please ensure that your manuscript meets PLOS ONE's style requirements, including those for file naming.*

We have thoroughly reviewed PLOS ONE's style guidelines and revised the manuscript accordingly.

This includes ensuring consistent formatting for headings, tables, figures, and references; using standard English; and adhering to file naming conventions (e.g., renaming supporting files to "S1_File.pdf" for the questionnaire and "S2_File.xlsx" for the dataset, as .sav files were problematic). Changes include updating reference citations to square brackets throughout, standardizing p-value formatting, and ensuring tables are editable and properly labeled. The manuscript now fully complies with PLOS ONE's requirements.

*2. In the ethics statement in the Methods, you have specified that verbal consent was obtained. Please provide additional details regarding how this consent was documented and witnessed, and state whether this was approved by the IRB.*

We have expanded the ethics statement in the Methods section to include these details. Specifically, we added: " All study procedures adhered to the principles of the Declaration of Helsinki. Participation was voluntary, and verbal informed consent was obtained at the beginning of each telephone interview, as the survey format rendered written consent impractical. Prior to consent, interviewers clearly explained the voluntary nature of participation, the right to withdraw at any time, and the measures taken to ensure confidentiality and anonymity. These measures included obtaining phone numbers from the Health and Treatment Deputies of Tehran University of Medical Sciences (TUMS) without any accompanying personally identifiable information and administering all questionnaires anonymously. Verbal consent was documented by the interviewer in a secure log, recording the date, time, participant’s anonymized identification code, and confirmation of consent. The TUMS Ethics Committee reviewed and approved this consent procedure as appropriate for the study.."

This addresses the need for transparency on documentation and IRB approval.

*3. Thank you for stating the following financial disclosure: “This study was supported in part by funding from Tehran University of Medical Sciences as a student thesis grant.” Please state what role the funders took in the study. If the funders had no role, please state: "The funders had no role in study design, data collection and analysis, decision to publish, or preparation of the manuscript." If this statement is not correct you must amend it as needed. Please include this amended Role of Funder statement in your cover letter; we will change the online submission form on your behalf.*

**Response:** We have amended the financial disclosure in the cover letter to include: "*This study was supported in part by funding from Tehran University of Medical Sciences as a student thesis grant. The funder had no role in study design, data collection and analysis, decision to publish, or preparation of the manuscript.”* This text has been highlightd in blue. This accurately reflects the funder's involvement. Unfortuantely, we did not find a location to include this statement in the submission system.

*4. We are unable to open your Supporting Information file [S2 File.sav]. Please kindly revise as necessary and re-upload.*

**Response:** We apologize for the issue. The .sav file (SPSS format) was not accessible, so we have converted the anonymized dataset to an editable .xlsx format (Excel) for broader compatibility. The file has been renamed "S2_File.xlsx" per PLOS ONE guidelines and will be re-uploaded with the revision. No data were altered during conversion.

**Reviewer #1 Comments**

Dear reviewer, we would like to begin addressing your comments by noting that we have now made our data file accessible via converting it into a excel format. Thus, **all** data on which our manuscript relies have been provided in the study and this suporting file.

*Overall Assessment: The reviewer noted the manuscript is interesting and intelligible but requires revisions and clarifications.*

We are very thankful for this kind evaluation and have made targeted changes to address shortcomings.

*Title:*

1- *Please add type of study.*

To clarify the design upfront and align with journal norms for transparency, we have revised the title to explicitly include the study type: "Parents’ perspectives on a national child oral health promotion program: sociodemographic influences and behavioral insights – a cross-sectional study."

*Abstract:*

2- *Please stick to Journal style.*

We have reformatted the abstract to match PLOS ONE's structured style (Background, Methods, Results, Conclusions) and ensured uniform formatting (e.g., p-values as "p=0.040").

3- *Please revise for uniform Journal style, see "p = 0.040".*

Consulted some recent PLOS one publications, All p-values in the abstract (and throughout the manuscript) have been standardized to "p = 0.040" without inconsistencies. We stuck to using the lowercase p, at least two decimal points of reporting, consistent spacing, and omitted leading zeros in all cases.

4- *When it comes to your conclusions, please exclusively stick to your aims. Remember that you wanted To "explore parents’ perceptions of Iran’s SOHPP, the sociodemographic factors shaping them, and children’s post-program oral health behaviors". Do not simply repeat your results here. Do not speculate. Do not provide well-accepted (but meaningless) phrases. Instead, provide a reasonable and generalizable extension of your outcome.*

**Response:** The abstract Conclusions section (and the manuscript conclusions section) have now been revised to focus on extensions of the findings tied to the aims, including recommendations for program improvement based on perceptions and sociodemographics, without repetition or speculation:

*Intro*

5- *More than two full pages would not be adequate for such a study, Please simply elaborate both aims and objectives, and present a sound rationale. A one-page Intro would seem adequate.*

**Response:** We appreciate the reviewer’s guidance regarding proportionality and concision.

In line with this comment, we undertook a full restructuring of the Introduction to remove non-essential global contextual material, compress epidemiologic background, eliminate duplicative rationale text, and focus the narrative on the SOHPP, the evidence gap, and the justification for the present cross-sectional analysis.

However, we were unfortuanely not able to compress the Introduction to a strict one-page limit, due in part to the need to (i) briefly characterize the national SOHPP architecture for readers unfamiliar with Iran’s health-system context and (ii) meet another reviewer’s comprehensive requests for a clearer articulation of the study rationale and existing research gaps at the intrentaional, regional, and national levels. Still we have reduced the section substantially compared to the previous submission to onlycontains core elements: a concise problem statement, a focused rationale, a comprehensive gap statement, and a clear and explicit statement of aims and objectives. We trust that this revision meets the intent of this comment.

*6- Please adapt your reference style, and use square brackets. "(...) of school-aged children worldwide (1, 2)." must read "(...) of school-aged children worldwide [1, 2]." Revise thoroughly.*

All citations have been changed to square brackets throughout the manuscript (e.g., [1, 2]).

*7- At the end of this section, please remember that you have statistically analyzed your data, so, consequently, a sound null hypothesis must be presented. The latter must be deducible from the foregoing thoughts.*

**Response:** We added a null hypothesis at the end of the Introduction: "We hypothesized that there would be no significant differences in parents' perceptions of the SOHPP or children's post-program oral health behaviors based on sociodemographic factors."

*Methods:*

*8- Please remember that Plos One will consider publishing qualitative research only if it adheres to appropriate study design and reporting guidelines... You have submitted the outcome of "phone-surveyed parents of primary school children", and such a study might be semi-quantitative, or even qualitative. Please clarify.*

**Response:** We thank the reviewer for emphasizing this important point and giving us the opportunity to clarify our study design. This study is a **quantitative cross-sectional analytical survey** and **does not contain any** qualitative or mixed-methods component.

- All data were collected using a structured questionnaire consisting almost entirely of closed-ended items (yes/no/no opinion, predefined frequency categories, and single-item ratings)
- The four principal outcome measures (overall awareness score, overall satisfaction score, acceptance of fluoride therapy, and perceived impact on tooth-brushing) were derived numerically (range 0–8 or binary).
- One open-ended item on school food was immediately post-coded into a dichotomous variable (healthy/unhealthy) according to predefined WHO criteria and analyzed quantitatively.
- No transcripts, quotations, themes, or interpretive coding were generated at any stage.
- No responses were subjected to thematic or content analysis, and no textual quotations are presented.
- Statistical methods were exclusively quantitative (descriptive statistics, ANOVA, chi-square tests, multiple linear regression, and binary logistic regression).
- Sample size was determined by power calculation for proportion estimation, not thematic saturation.

Therefore, and since our study fully complies with standard reporting expectations for quantitative observational research (STROBE guidelines), it does **not** require qualitative reporting checklists such as COREQ or SRQR.

To eliminate any possible ambiguity, we have added the following sentence in the first paragraph of the Methods section: *“This is a quantitative cross-sectional analytical study based on a structured telephone survey; no qualitative or mixed-methods data collection or analysis was performed.”*

**Response:** We added a discussion in the Sample Size subsection: "Our sample size (n=354) aligns with recommendations for quantitative surveys in health research [reference to the cited article], providing adequate power for detecting differences in perceptions while balancing feasibility in a telephone-based setup."

9- *Cross-sectional studies are a type of observational studies. The latter must be pre-registered... Please provide you a-priori registration date and number.*

**Response:** We thank the reviewer for emphasizing the importance of transparency and a-priori planning in observational research. We fully agree that preregistration represents best practice in many research contexts and is mandatory for randomized controlled trials.

However, we would like to respectfully clarify that the present study is a **cross-sectional analytical observational** study, and is **not** a propsective or retrospective observational study. Under current international reporting standards for observational research, including the STROBE guidelines, **preregistration is not a formal requirement for cross-sectional study designs.** Rather, mandatory registration applies to randomized controlled trials and some prospective observational studies.

This study was nonetheless conducted according to an a-priori protocol submitted as a proposal addressing the Tehran University of Medical Sciences. The submission of such propsal was neessarry for receiving an ethical approval from the Tehran University of Medical Sciences Ethics Committee (which this study did, with the code: IR.TUMS.DENTISTRY.REC.1399.011) prior to data collection. The study objectives, sampling strategy, measurement approach, and statistical analysis plan were defined before data collection and are transparently reported in the Methods section. In addition, the questionnaire and anonymized dataset are provided as supplementary materials to further support transparency.

*10 - Please go to https://journals.sagepub.com/doi/10.1177/1049732315617444, and discuss your set-up with reference to your sample size.*

**Response:** We thank the reviewer for directing us to this insightful reference and their guidance, hepling strengthen the methodological transparency of our manuscript.

The cited article by Malterud et al. (2016) proposes the concept of “information power” as a pragmatic alternative to “saturation” for guiding sample size determination in qualitative interview studies. The authors emphasize that information power, the relevance and density of data from participants, varies based on factors such as study aim, sample specificity, use of established theory, quality of dialogue, and analysis strategy, allowing for smaller samples when information is rich and targeted.

However, as clarified in our response to Comment 8, the present study is **not** a qualitative or mixed-methods study. It is a **quantitative cross-sectional survey** employing a short (approximately 10–12 minute), fully structured, checklist-based telephone questionnaire with predominantly closed-ended items (see Supporting File S1). No semi-structured or in-depth interviews were conducted, and data were not analyzed for thematic depth, emergent insights, or narrative richness. Instead, all outcomes were quantified (e.g., composite scores 0–8, binary/ordinal categories) and subjected to parametric and non-parametric statistical tests (ANOVA, chi-square, regression). Sample size was therefore determined using established quantitative methods: a pilot-based single proportion formula in SPSS (α = 0.05, expected proportion p = 0.69 from pilot data, margin of error d = 0.10), yielding a minimum required n = 347 (achieved n = 354 with 67% response rate). Concepts such as information power or saturation, which apply to qualitative designs aiming for interpretive depth, are not relevant to our structured survey approach.

To further clarify, we have added the following sentence in the Methods → Sample size calculation subsection: *“following the standard sample size calculation pathway for quantitative proportion-based surveys,…”*

*11-Your sample size calculation would not seem clear. Please provide more details. See comments given above.*

**Response:** We thank the reviewer for this valuable comment and for the opportunity to provide greater transparency regarding our sample size determination.

As now explained and clarified in our responses to comments 8 and 10 above and 12 below, this is a **quantitative cross-sectional analytical survey** employing a structured, closed-ended telephone questionnaire, with all outcomes quantified and analyzed using standard inferential statistics. Sample size was therefore calculated using established quantitative methods for estimating a single proportion with specified precision, rather than qualitative concepts such as information power or saturation (Malterud et al., 2016). Specifically, the calculation was carried out using α = 0.05, p = 0.69, and d = 0.1. This yielded a minimum required sample size of **n = 347.** We successfully recruited and obtained complete data from **n = 354** participants (exceeding the minimum), achieved after approaching 525 eligible parents to account (response rate of approximately 67%).

*12- With your calculated sample size being 347, why did you approach "525 individuals"? Please clarify.*

**Response:** We thank the reviewer for highlighting this point and providing the opportunity to clarify our recruitment process. The minimum required sample size was calculated as n = 347 (as detailed in our response to comment 11). However, anticipating potential non-response, a common challenge in telephone-based surveys, we proactively oversampled to ensure the target was met or exceeded. In total, 525 eligible parents were approached. Of these, 354 provided complete responses, yielding an effective response rate of approximately 67% (354/525). This achieved sample size (n = 354) slightly exceeded the calculated minimum, enhancing statistical power for the planned subgroup analyses while remaining feasible within the study's resources.

To improve transparency, we have revised the relevant sentence at the very beginning of Results section as follows:

"To reach the minimum required sample size while accounting for anticipated non-response, a total of 525 individuals were approached, ultimately resulting in 354 participants (a 67% response rate). "

*13- Same with " Four centers, Ayat, Farmafarmanian, Imam Hassan Mojtaba, and Avicenna, were randomly selected from this list." Please clarify your randomization approach.*

**Response:** We thank the reviewer for this comment and agree that greater detail on the randomization process enhances reproducibility. To ensure unbiased selection, the full list of centers was entered into a spreadsheet (Microsoft Excel; Microsoft Corporation, Redmond, WA, USA). A random number was generated for each center using Excel’s RAND() function, and the list was sorted by these values. The top four centers, Ayat, Farmafarmanian, Imam Hassan Mojtaba, and Avicenna, were then selected. We have revised the text in the Methods section to reflect these undertaken action.

*13- Note that with ALL materials and methodologies (including statistical software), please use general/non-proprietary names with your text, followed by (brand name; manufacturer, city, ST[ate - abbreviated, if US], country) in parentheses. Stick to semicolon. Revise thoroughly.*

**Response:** We have revised the manuscript to ensure that all materials, software, and methodological tools are reported using general/non-proprietary terminology, followed by the proprietary name, manufacturer, city, state (where applicable), and country in parentheses, separated by semicolons.

*Results*

*14- Again, compare "p = 0.040", "p = .011", "p = 0.040" and "P-value". Revise for uniform Journal style. Consulting some recently published Plos One papers should be helpful.*

**Response:** All p-values standardized to "p=0.040" format, consistent with recent PLOS ONE articles.

*Discussion*

*15- Stick to H0 when starting this section.*

**Response:** We would like to respectfully clarify that the explicit null-hypothesis statement in the Introduction was added in response to an earlier reviewer request and was intentionally framed to apply **only to the study’s inferential objectives**. Accordingly, the Discussion begins with a descriptive and integrative summary of the main findings related to parental perceptions and reported behaviors, which correspond to both descriptive and inferential aims of the study. Reference to the null hypotheses is then introduced later in the opening paragraph, where the inferential results are interpreted and the partial rejection of the null hypotheses is reported. We believe this structure maintains consistency with the reviewer’s requests while adhering to standard methodological practice, whereby null-hypothesis framing is restricted to inferential analyses rather than descriptive synthesis.

*16- Do not provide a literature review here. This section will benefit from a sound DISCUSSION.*

**Response:** We thank the reviewer for this constructive and helpful comment. We have now revised the Discussion to be explicitly result-driven, reducing descriptive literature review elements and reorienting the section toward interpretation of our findings, contextual explanation, and programmatic implications, while retaining only those external comparisons necessary to contextualize our results.

*Conclusions*

*17- This section would not seem satisfying. Remember that "Conclusions" does not mean "Summary" (or "Repetition of Outcome"). Revise carefully.*

*and*

*18- Again, with your Conclusions, please stick exclusively to your aims. Do not simply repeat your results here. Do not speculate. Do not provide well-accepted (but meaningless) phrases. Instead, provide a reasonable and generalizable extension of your outcome.*

**Response:** We thank the reviewer for the guidance. The Conclusion has been fully revised to avoid repetition of results and to remain tightly linked to the study aims. The revised text no longer summarizes outcomes; instead, it interprets parental perceptions in terms of actionable implications for the program. Also, the current paragraph does not speculate beyond the dataset and provides a concise, generalizable extension of the findings relevant to SOHPP refinement.

*References*

*19- Your references list must be adapted to Journal style. Remember that this is considered your task, to avoid any mistakes.*

**Response:** References reformatted to PLOS ONE style (Vancouver-like, with DOI where available). Checked for accuracy.

*In total, several shortcomings and drawbacks do not allow for any proceeding with this draft, and re-review is considered mandatory.*

We have strved to address all of your raised points comprehensively and believe the revisions resolve these issues. Thank you again for your attention to detail and comprehensive feedback.

**Reviewer #2 Comments**

1- *The introduction could be strengthened by more clearly outlining why parents’ perspectives are critical for the long-term success and sustainability of school-based oral health programs.*

**Response:** We thank the reviewer for this valuable suggestion. In response, we have revised the Introduction to more explicitly position parents as central stakeholders in child oral health promotion. The revised text now clarifies how parents shape children’s oral health knowledge, attitudes, and behaviors, and why their engagement is essential for effective program delivery, uptake, and long-term sustainability. We further highlight evidence showing that programs with active parental involvement achieve stronger outcomes, and we reference international guidance recognizing parental engagement as a prerequisite for durable and equitable chil oral health promotion interventions.

2- *It would also help to clarify how this study addresses existing gaps in the regional or international literature.*

**Response:** We appreciate this important point. The Introduction has been expanded to more clearly delineate gaps in the existing literature at international, regional, and national levels. Specifically, we now note that prior evaluations of school-based oral health programs have predominantly focused on children’s clinical or behavioral outcomes, with parents’ perspectives assessed far less consistently, often through small, context-specific qualitative studies in high-income settings. We further highlight the scarcity of evidence from the Middle East and Iran, particularly regarding national programs and component-level parental assessments. The revised text explicitly situates the present study as addressing these gaps by providing a comprehensive, component-level evaluation of parents’ perceptions of Iran’s national SOHPP and examining sociodemographic correlates of these perceptions.

3- *Regarding questionnaire validation, the manuscript mentions face and content validity and a test–retest approach. Including additional information—such as the time interval between tests or any calculated reliability values—would make the instrument’s credibility clearer.*

**Response:** We fully agree that providing more detail about the validation process strengthens the transparency and credibility of the study instrument. Accordingly, we have substantially revised the relevant paragraph in the “Study tool and measures” section to include the following additional information:

- Face and content validity were assessed by group discussions among **five faculty experts** in Community Oral Health at Tehran University of Medical Sciences (TUMS), who reviewed the items for relevance, clarity, and comprehensiveness over **iterative rounds**.
- The test-retest reliability procedure was conducted with a subsample of **20 parents**, with the retest administered **two weeks** after the initial test to balance response stability and minimization of recall bias.
- Given the preliminary and iterative purpose of this validation step (and the checklist-based, interviewer-administered nature of the tool), the focus was on qualitative identification of inconsistencies and ambiguities rather than computation of formal statistical reliability coefficients (e.g., ICC or Cohen’s kappa).
- Informal review of the paired responses showed **high consistency** across most items (**approximately 85% agreement**), and only minor revisions were made to improve item wording and instruction

4- *Because the awareness and satisfaction indices were based on only a few items, it might be worth acknowledging this as a methodological limitation. You could suggest that future studies use a more detailed psychometric assessment, such as reliability testing or factor analysis, to improve measurement precision.*

**Response:** We thank the reviewer for this helpful methodological observation. We have now explicitly acknowledged in the Limitations section that the awareness and satisfaction indices were based on a number of checklist-style items targeting specific program components, which may limit measurement precision. We clarify that this approach was chosen deliberately for a pragmatic, interviewer-administered evaluation of SOHPP delivery rather than for measuring broader latent constructs. We also note that future studies could employ more comprehensive psychometric instruments, including multi-item Likert scales or factor-analytic assessments, to enhance measurement depth and precision

5- *The statistical approach is well chosen and explained. However, the use of backward regression could be better justified. A brief explanation of why this method was selected, and whether sensitivity analyses were done to confirm model stability, would enhance transparency.*

**Response**: We appreciate the reviewer's positive assessment of the statistical approach and the helpful suggestion for added transparency.

Upon re-checking our analysis logs in response to this comment, we identified an oversight in the manuscript description: the multiple linear and binary logistic regression models were actually fitted using the **enter** method (forcing all sociodemographic predictors into the models simultaneously), rather than backward stepwise elimination as stated. This was a textual error during manuscript preparation, and we apologize for this oversight and any confusion it may have caused. Importantly, the error does not affect the reported results, which are based on the full models (consistent with the enter approach, retaining all predictors regardless of significance). We have corrected the description in the Statistical Analysis section of the revised manuscript to accurately reflect the enter method. The enter method was selected as it is the standard and default approach in regression analysis, particularly appropriate for exploratory studies like ours where there are no strong a priori hypotheses about the relative importance of specific sociodemographic predictors. Given the exploratory nature of this research, aiming to comprehensively examine potential associations across a set of sociodemographic variables rather than to test confirmatory hypotheses, the enter method ensures unbiased estimation of effects for all included variables while mutually adjusting for their shared variance.

In addition, we agree that sensitivity analyses are valuable tools for assessing model robustness in many analytical contexts. In the present study, however, formal sensitivity analyses were not conducted because they were **not methodologically appropriate given the study design, objectives, and data structure**. This was a **cross-sectional, exploratory observational study** with hypothesis-generating aims rather than causal inference or prediction. Sensitivity analyses are most informative when models rely on strong assumptions. In our study, the primary outcomes were **directly observed, checklist-based variables**, derived from clearly defined questionnaire items rather than latent constructs or modeled exposures. As such, there were no plausible alternative exposure or outcome definitions to test. In addition, the regression models were **intentionally parsimonious**, specified a priori based on the study objectives and sample size considerations. The number of covariates was limited, no interaction terms or complex modeling strategies were employed, and the Enter method was used consistently to avoid data-driven model specification. Under these conditions, conducting multiple alternative model specifications would not meaningfully improve interpretability and could instead introduce unnecessary analytical flexibility. Finally, instead of formal sensitivity analyses, we assessed **model stability and robustness**. These included evaluation of regression assumptions (normality, linearity, and homoscedasticity), and assessment of multicollinearity using variance inflation factors (all VIFs < 5). These checks indicated stable estimates across all fitted models.

6- *The discussion is well developed and connects effectively with previous research. Still, the paper would benefit from a stronger emphasis on practical implications—particularly how the observed links between fathers’ education, government employment, and program perceptions could guide the design of future oral health interventions or communication strategies.*

**Response:** We thank the reviewer for this constructive suggestion. In response, we have substantially strengthened the Discussion by explicitly translating the observed associations into concrete, actionable implications for program design and communication. Specifically, we now (i) link higher fathers’ education to differentiated communication strategies; (ii) propose workplace-based outreach strategies tailored to households headed by government employees; and also (iii) outline practical measures to address procedural transparency concerns underlying fluoride therapy refusal. These additions clarify how the study’s sociodemographic findings can directly inform the refinement and targeting of the SOHPP (and comparable LMIC programs) in the future.
